# Hormetic Antioxidant Effects of *Uncaria gambir* on Male Reproductive Function: A Comprehensive Experimental and Computational Analysis

**DOI:** 10.3390/ijms27177533

**Published:** 2026-08-22

**Authors:** Hendri Devita, Idris Adewale Ahmed, Yudha Endra Pratama, Maryam Abimbola Mikail

**Affiliations:** 1 Department of Biotechnology, School of Health and Applied Science, Lincoln University College, No. 2, Jalan Stadium SS7/15, Kelana Jaya, Petaling Jaya 47301, Selangor, Malaysia; hendridevita@jurkeb.unbrah.ac.id (H.D.); ajike1404@gmail.com (M.A.M.); 2 Midwifery Program, Faculty of Health Science, Baiturrahmah University, Padang 25586, West Sumatra, Indonesia; 3 Centre for Probiotic Biotechnology, Baiturrahmah University, Padang 25586, West Sumatra, Indonesia; yudhaendra.pratama@gmail.com

**Keywords:** *Uncaria gambir*, catechin, antioxidant, antimicrobial, testosterone

## Abstract

Oxidative stress is a key mechanism underlying nicotine-induced male reproductive dysfunction, yet evidence-based phytopharmacological interventions remain limited. *Uncaria gambir* Roxb., a catechin-rich medicinal plant from West Sumatra, Indonesia, exhibits strong antioxidant potential; however, a comprehensive multi-regional evaluation combined with in vivo androgenic assessment is lacking. This study aimed to characterize the antioxidant activity, phytochemical composition (HPLC-DAD), and antimicrobial properties of gambir extracts from three production regions (Halaban, Mungka, and Pesisir Selatan), to evaluate catechin pharmacokinetics in silico (PASS Online; SwissADME), and to assess dose-dependent effects in male Wistar rats exposed to nicotine (n = 36). The Halaban extract showed the highest antioxidant activity (IC_50_ = 6.99 ± 0.28 µg mL^−1^) and catechin content (7.91 ± 0.07 mg g^−1^). All extracts demonstrated broad-spectrum antimicrobial activity. PASS analysis predicted strong membrane integrity agonism (Pa = 0.950) and HMOX1 induction (Pa = 0.778), while SwissADME indicated favorable pharmacokinetic properties, including full Lipinski compliance and high gastrointestinal absorption. In the in vivo study, gambir Halaban extract at 300 mg kg^−1^ day^−1^ significantly increased follicle-stimulating hormone, luteinizing hormone, and testosterone levels compared to controls (*p* < 0.05), exceeding baseline values and indicating HPG axis stimulation. A non-linear (hormetic) response was observed, with reduced efficacy at higher doses. These findings highlight bioactive-rich gambir as a promising multi-target natural antioxidant for mitigating oxidative-stress-related reproductive dysfunction, warranting further mechanistic and translational studies.

## 1. Introduction

Oxidative stress, characterized by a systemic imbalance between the production of reactive oxygen species (ROS) and the capacity of antioxidant defenses, is increasingly recognized as a core pathophysiological mechanism in various non-communicable diseases, including cardiovascular disorders, metabolic syndrome, neurodegenerative diseases, and male infertility [1,2]. Reactive oxygen species, including superoxide anion (O_2_•−), hydrogen peroxide (H_2_O_2_), and the highly reactive hydroxyl radical (•OH), have deleterious effects on biological macromolecules such as DNA, proteins, and membrane phospholipids via peroxidative chain reactions. Under physiological conditions, endogenous antioxidant systems such as superoxide dismutase (SOD), glutathione peroxidase (GPx), and catalase effectively neutralize ROS. However, environmental toxins, nutritional deficiencies, and lifestyle factors can overwhelm these defenses, leading to chronic oxidative damage [1,2].

Male reproductive tissues are particularly susceptible to oxidative damage due to the elevated polyunsaturated fatty acid (PUFA) content in the sperm plasma membrane, making spermatozoa especially prone to structural and functional impairment from lipid peroxidation. Nicotine, the primary addictive alkaloid in tobacco, induces oxidative stress via many mechanisms, including disruption of the mitochondrial electron transport chain, activation of NADPH oxidase by catecholamines, and direct generation of pro-oxidant metabolites. Chronic nicotine exposure is linked to inhibited hypothalamic–pituitary–gonadal (HPG) axis function, resulting in decreased serum gonadotrophins (follicle-stimulating hormone and luteinizing hormone) and testosterone, impaired spermatogenesis, and structural damage to spermatozoa. The worldwide prevalence of male factor infertility, which accounts for roughly 50% of infertility cases globally, alongside the increasing tobacco use in developing countries, highlights the pressing necessity for accessible, evidence-based strategies aimed at mitigating nicotine-induced reproductive oxidative damage [3,4,5].

Phytochemical antioxidants, especially polyphenols, have garnered ongoing scientific interest as supplementary or alternative medicinal agents because of their diverse modes of action, favorable safety profiles, and wide availability. Catechin ((2R,3S)-2-(3,4-dihydroxyphenyl)-3,4-dihydro-2H-chromene-3,5,7-triol), a predominant flavan-3-ol among naturally occurring polyphenols, demonstrates antioxidant properties via hydrogen atom transfer (HAT) and single electron transfer (SET) mechanisms facilitated by its catechol B-ring ortho-dihydroxyl group. In addition to direct radical scavenging, catechin regulates intrinsic cytoprotective mechanisms, including nuclear factor erythroid 2-related factor 2 (Nrf2)/haem oxygenase-1 (HMOX1) signaling, suppresses pro-inflammatory NF-κB pathways, chelates redox-active metal ions, and mitigates lipid peroxidation. These mechanisms suggest that catechin-rich botanical extracts may mitigate reproductive endocrine disruption caused by oxidative stress induced by nicotine [1,6,7,8,9].

Gambir (*Uncaria gambir* Roxb.; family Rubiaceae) is a climbing shrub native to Southeast Asia that holds significant economic value in Indonesia, especially in West Sumatra, which contributes almost 80% of the national output [10,11]. The concentrated extract of gambir derived from the leaves and young stems of *U. gambir* is notably abundant in catechin (often reported at 22–50% of dry weight), condensed tannins, gallic acid, quercetin (flavonoid aglycone), and rutin (flavonoid glycosides) [12,13,14]. Pharmacological studies have established the antioxidant, antibacterial, anti-inflammatory, antiulcer, and gastroprotective properties of gambir extracts [11,15,16]. However, a comprehensive multi-regional quality benchmarking of West Sumatran gambir that incorporates phytochemical quantification, antioxidant profiling, antimicrobial screening, and in silico computational analysis alongside in vivo reproductive endocrine assessment has not been conducted.

A significant research deficiency exists at the convergence of gambir phytochemistry and male reproductive pharmacology. While several studies have recorded the antioxidant activity of gambir extracts from Indonesian sources [15,17,18,19], the variability due to geographic origin, agronomic circumstances, and post-harvest processing has seldom been comprehensively examined using HPLC-DAD quantification [17]. Moreover, no research thus far has assessed the pharmacokinetic drug-likeness of gambir catechin using contemporary computational methods (PASS Online and SwissADME) alongside in vivo evaluation of its androgenic effects in a nicotine-induced model of male reproductive oxidative stress.

This study aims to: (i) characterize the antioxidant capacity and HPLC-DAD phytochemical composition of gambir extracts from three production regions in West Sumatra; (ii) evaluate broad-spectrum antimicrobial activity; (iii) predict the biological activity spectrum and pharmacokinetic profile of catechin using PASS Online and SwissADME in silico tools; and (iv) assess the in vivo protective effects of varying doses of gambir extract on serum follicle-stimulating hormone (FSH), luteinizing hormone (LH), and testosterone levels in nicotine-challenged male Wistar rats. This integrated in vitro, in silico, and in vivo investigation establishes a scientifically robust basis for the advancement of gambir catechin as a multi-target natural antioxidant therapy.

## 2. Results

### 2.1. Antioxidant Activity and Phytochemical Composition

The antioxidant potency and HPLC-DAD-quantified phytochemical composition of gambir extracts from three West Sumatran production regions are summarized in Table 1. One-way ANOVA identified statistically significant inter-regional differences in DPPH IC_50_ values, catechin content, and condensed tannin content (*p* < 0.05 for all parameters).

The three gambir extracts showed estimated DPPH IC_50_ values ranging from 6.99 to 9.50 µg mL^−1^. The Halaban extract exhibited the lowest IC_50_ (6.99 ± 0.28 µg mL^−1^), followed by Pesisir Selatan (8.49 ± 0.07 µg mL^−1^) and Mungka (9.50 ± 0.13 µg mL^−1^). These values were estimated from concentration–response data using nonlinear logistic regression within the tested concentration ranges.

### 2.2. HPLC-DAD Phenolic and Flavonoid Profile

Qualitative HPLC-DAD analysis was performed to examine selected phenolic- and flavonoid-related chromatographic features in gambir extracts from Halaban, Mungka, and Pesisir Selatan. The reference standards showed retention times of 5.746 min for gallic acid, 14.695 min for quercetin, and 11.731 min for rutin under the applied analytical conditions. Comparison of the sample chromatograms with the corresponding reference-standard chromatograms did not show consistent retention-time correspondence for any of the three compounds. The sample extracts showed several chromatographic peaks, with prominent signals occurring at approximately 1.9–2.1 and 3.0–3.1 min. Additional peaks were observed at approximately 13.83–13.85 min in extracts from all three production regions. For example, representative chromatograms showed peaks at 1.976, 3.042, 9.389, and 13.834 min in the Halaban extract, while peaks at 1.976, 3.060, 9.428, and 13.843 min were observed in the Mungka extract.

Because these sample peaks did not consistently correspond to the retention times of the respective reference standards, the observed chromatographic signals could not be conclusively assigned to gallic acid, quercetin, or rutin. The HPLC-DAD findings are therefore considered a qualitative assessment of selected chromatographic features rather than definitive identification of individual phenolic or flavonoid compounds. Representative chromatograms of the reference standards and gambir extracts from the three production regions are provided in the Supplementary Materials (Supplementary Figures S1 and S2).

### 2.3. Pearson Correlation Between Phytochemical Content and Antioxidant Activity

The Pearson correlation analysis between phytochemical content and antioxidant IC_50_ values is presented in Table 2.

### 2.4. Antimicrobial Activity

Inhibition zone diameters (mm) against six reference strains are presented in Table 3.

### 2.5. PASS Biological Activity Prediction

PASS-predicted biological activity probabilities for catechin are presented in Table 4.

PASS prediction identified catechin as exhibiting high-probability activities across membrane integrity agonism (Pa = 0.950), HMOX1 expression enhancement (Pa = 0.778), TP53 upregulation (Pa = 0.826), caspase-3 stimulation (Pa = 0.761), and lipid peroxidase inhibition (Pa = 0.692), alongside moderate antioxidant (Pa = 0.587) and free radical scavenging (Pa = 0.633) activities.

### 2.6. Pharmacokinetic Profile of Catechin (SwissADME)

The SwissADME-predicted pharmacokinetic and drug-likeness parameters of catechin are summarized in Table 5.

### 2.7. In Vivo Androgenic Effects: HPG Axis Hormones

Serum concentrations of FSH, LH, and testosterone across experimental groups following 30-day treatment are presented in Table 6. One-way ANOVA identified statistically significant inter-group differences in FSH (F (5,30), *p* = 0.006), LH (*p* = 0.037), and testosterone (*p* = 0.001).

## 3. Discussion

All three gambir extracts demonstrated potent DPPH radical scavenging activity, with IC_50_ values ranging from 6.99 to 9.50 µg mL^−1^, substantially below the 200 µg mL^−1^ threshold conventionally considered indicative of strong plant-derived antioxidant activity [20,21]. The Halaban extract exhibited the lowest IC_50_ (6.99 ± 0.28 µg mL^−1^), indicating the greatest free-radical scavenging capacity, while the Mungka extract exhibited the weakest potency (IC_50_ = 9.50 ± 0.00 µg mL^−1^). These values are consistent with the literature [18] (IC_50_ 7.8–12.4 µg mL^−1^), indicating strong DPPH-scavenging activity under the experimental conditions in the published literature. Importantly, the IC_50_ values obtained in the present study are also comparable to and, in the case of Halaban, superior to synthetic antioxidants such as butylated hydroxytoluene (BHT; IC_50_ ≈ 12–18 µg mL^−1^) under equivalent DPPH assay conditions [22], underscoring the antioxidant potential of this plant.

The superior antioxidant capacity of the Halaban extract could be mechanistically attributable to its highest catechin content (7.91 ± 0.07 mg g^−1^). The nonlinear concentration–response analysis confirmed strong antioxidant activity across the three gambir extracts, with the Halaban extract showing the lowest IC_50_. This finding is consistent with its comparatively higher catechin content, suggesting that the greater antioxidant activity of the Halaban extract may be related, at least in part, to its richer catechin composition. Catechin, the principal flavan-3-ol constituent of gambir, exerts DPPH radical scavenging activity primarily through the ortho-catechol (3′,4′-dihydroxyl) moiety of its B-ring via two complementary mechanisms: hydrogen atom transfer (HAT), in which the phenolic O−H bond dissociation enthalpy governs proton-coupled electron transfer to the DPPH radical, and single electron transfer (SET), in which direct electron donation generates the more stable catechin semiquinone radical [23,24]. The standard electrode potential of the catechol/semiquinone redox couple (E°′ ≈ 0.50 V) is thermodynamically favorable relative to the DPPH•/DPPH− couple (E°′ ≈ 0.71 V), providing the electrochemical driving force for SET [23]. Furthermore, the resulting semiquinone radical is resonance-stabilized across the aromatic B-ring system, a structural feature that disfavors radical chain propagation and thereby enhances effective antioxidant stoichiometry [25]. These mechanistic considerations explain why catechin content, more so than condensed tannin content, governs overall DPPH scavenging capacity in the present dataset.

The pronounced inter-regional variation in catechin content (3.59–7.91 mg g^−1^) reflects a complex interplay of pedoclimatic and post-harvest factors. Altitude-dependent UV-B irradiance drives differential flavonoid biosynthesis via the phenylalanine ammonia-lyase (PAL) pathway; higher-altitude cultivation zones in Limapuluh Kota Regency (Halaban and Mungka districts, ~500–800 m a.s.l.) relative to the coastal Pesisir Selatan region are associated with enhanced catechin accumulation through transcriptional upregulation of PAL and chalcone synthase (CHS) enzymes [11,17]. Post-harvest processing parameters, particularly the duration and temperature of traditional pressing and sun-drying, additionally modulate catechin content through thermal oxidation and enzymatic polymerization into condensed proanthocyanidins [17,18]. The comparatively higher condensed tannin content in Halaban and Mungka samples (14.31–14.45 mg g^−1^ vs. 10.41 mg g^−1^ in Pesisir Selatan) indicates greater polycondensation, yet this was not associated with proportionally enhanced DPPH inhibition. This observation is consistent with the established principle that high-molecular-weight condensed tannins (proanthocyanidins) exhibit reduced diffusivity toward DPPH radicals in methanol solution owing to steric constraints that limit effective radical–polyphenol encounters [26]. These findings reinforce the necessity of standardised HPLC-DAD catechin quantification as a mandatory quality benchmark for pharmaceutical-grade gambir.

The qualitative HPLC-DAD profiles observed across the three gambir extracts are consistent with the complex polyphenolic composition previously reported for *Uncaria gambir*, which includes gallic acid, epicatechin, quercetin, kaempferol, and procyanidin oligomers as secondary constituents alongside the dominant catechin fraction [11,27]. However, in the present study, gallic acid, quercetin, and rutin could not be conclusively assigned to the observed sample peaks because their retention times did not consistently correspond with those of the respective reference standards. The chromatographic profiles should therefore be interpreted as evidence of a complex phenolic and flavonoid fraction rather than as confirmation of these individual compounds. Because the present analysis was qualitative, the contribution of individual chromatographic constituents to the observed antioxidant activity could not be established. The antioxidant differences observed among the production regions are more appropriately considered in relation to the quantified catechin and condensed tannin contents, while the contribution of other phenolic constituents remains a possibility that requires further analytical confirmation.

Pearson correlation analysis revealed a moderate negative association between catechin content and IC_50_ (r = −0.489), directionally consistent with the expectation that higher catechin content confers superior DPPH radical scavenging potency. The negligible tannin–IC_50_ correlation (r = −0.082) reinforces the mechanistic interpretation that polymeric condensed tannins contribute minimally to small-molecule DPPH scavenging relative to monomeric catechin [26,28]. However, neither correlation attained statistical significance (both *p* > 0.05), an outcome that is mechanistically unsurprising given the critically underpowered dataset (n = 3 regional samples). With only three data points, the critical r-value at α = 0.05 (two-tailed) approaches ±0.997, rendering conventional hypothesis testing effectively meaningless and conferring undue leverage on individual observations. This critical limitation must be acknowledged unambiguously. A statistically well-powered multi-regional investigation incorporating ≥10 geographically and seasonally diverse West Sumatran gambir samples is warranted to definitively establish structure–activity relationships. Notwithstanding this limitation, the directional coherence of the catechin–IC_50_ relationship with established catechin pharmacology [7] and the corroborating HPLC-DAD data provide a scientifically plausible foundation for the hypothesis that catechin is the principal antioxidant determinant in gambir.

Catechin demonstrated full compliance with all four Lipinski Rule-of-Five criteria [29], establishing its membership within the oral drug-like chemical space. High predicted gastrointestinal absorption, confirmed by boiled-egg model visualization [30,31] is corroborated by clinical pharmacokinetic studies in humans that demonstrate peak plasma catechin concentrations of 0.1–0.5 µM within 1–2 h of a single oral dose of 170 mg catechin equivalents [32]. The consensus LogP of −0.48 reflects moderate hydrophilicity that facilitates rapid plasma distribution and renal excretion, consistent with the reported plasma elimination half-life of catechin (t_1_/_2_ ≈ 2–3 h in humans) [32] implying the necessity for multiple daily dosing in any future pharmaceutical or nutraceutical formulation targeting sustained antioxidant protection in male reproductive tissues.

The high TPSA (110.38 Å^2^) predicting limited blood–brain barrier penetration is pharmacologically advantageous for an agent targeting peripheral reproductive oxidative pathways, as it substantially reduces the probability of CNS off-target effects [33]. The complete absence of predicted inhibitory activity across the five major cytochrome P450 isoforms (CYP1A2, CYP2C19, CYP2C9, CYP2D6, CYP3A4) is of considerable clinical significance, indicating a low risk of pharmacokinetic drug–drug interactions [30], an important attribute given that male infertility patients frequently receive concurrent pharmacotherapy (e.g., hormonal supplementation, phosphodiesterase inhibitors).

The single PAINS alert (catechol moiety) was contextualized appropriately: although catechol-containing polyphenols are commonly flagged by computational PAINS filters due to their redox-cycling potential in biochemical assays [30], this structural feature constitutes the mechanistic basis for catechin’s validated antioxidant bioactivity; the catechol B-ring ortho-dihydroxyl moiety is precisely the pharmacophoric element responsible for HAT and SET radical scavenging, Keap1 cysteine modification, and Fe^2+^ chelation [7]. This interpretation is consistent with Baell [30], who explicitly cautions against categorical exclusion of catechol-containing natural products on the basis of PAINS flags, recognizing that redox activity can represent genuine target engagement rather than assay artefact. Collectively, the ADMET profile of catechin establishes a robust pharmacokinetic rationale for its in vivo investigation and supports its potential development as an orally bioavailable, target-selective natural antioxidant pharmaceutical.

Chronic nicotine exposure (NEG group) produced a numerically consistent trend toward HPG axis suppression relative to healthy controls (HCs), with reduced FSH (8.89 vs. 9.86 mIU mL^−1^) and testosterone (3.81 vs. 3.99 ng mL^−1^). Although these differences did not achieve statistical significance, likely attributable to the high intra-group variance in the NEG group (SD = ±2.79 for FSH, ±1.08 for testosterone), the directional trend is fully consistent with the extensively documented pro-oxidant mechanisms of nicotine on the HPG axis [34,35]. Nicotine and its primary metabolite cotinine suppress hypothalamic GnRH pulse amplitude through nicotinic acetylcholine receptor (nAChR)-mediated stimulation of noradrenergic pathways, reduce pituitary gonadotroph responsiveness to GnRH through ROS-mediated receptor downregulation, and directly impair testicular Leydig cell steroidogenesis via oxidative inactivation of CYP11A1 (P450scc) and the steroidogenic acute regulatory (StAR) protein [34]. The high intra-group hormonal variability in the NEG group is consistent with previously reported interindividual variation in nicotine metabolic rate and sensitivity in rat models [35] and underlines the importance of adequate group sizes in future studies.

A critical and unexpected finding was the significantly lower hormonal concentrations in the zinc sulphate positive control group (POS) compared with all gambir treatment groups and the healthy control, including the lowest testosterone values across all groups (2.69 ± 0.52 ng mL^−1^). This paradoxical hormonal suppression with high-dose zinc (100 mg kg^−1^ day^−1^) merits mechanistic consideration. While zinc is an essential cofactor for numerous testosterone biosynthetic enzymes, including 17β-hydroxysteroid dehydrogenase (17β-HSD) and 3β-hydroxysteroid dehydrogenase (3β-HSD), supraphysiological zinc supplementation induces competitive antagonism of copper absorption (Zn^2+^ upregulates intestinal metallothionein, sequestering Cu^2+^), resulting in copper deficiency-mediated impairment of cytochrome c oxidase activity and mitochondrial dysfunction in steroidogenic cells [34]. Furthermore, at supraphysiological concentrations, zinc ions can generate hydroxyl radicals through Fenton-type chemistry upon reaction with endogenous hydrogen peroxide, paradoxically exacerbating rather than attenuating testicular oxidative stress [34]. These data suggest that the dose of zinc sulphate employed (100 mg kg^−1^ day^−1^ in rats) substantially exceeds the physiologically beneficial range and is inappropriate as a positive control for androgenic supplementation. Future studies should employ a validated positive control such as clomiphene citrate (5 mg kg^−1^ day^−1^) or testosterone enanthate (10 mg kg^−1^ week^−1^) that has established HPG axis-stimulating activity in the nicotine-challenged rat model.

Admittedly, previous studies have reported beneficial effects of zinc supplementation on reproductive parameters, including sperm concentration, motility, and normal morphology. In male mice, zinc administration at doses of 10, 20, and 30 mg was associated with improvements in sperm parameters, with a dose-related increase observed across the treatment groups [34]. Other experimental studies have also evaluated zinc at doses up to 100 mg kg^−1^ in models of testicular oxidative stress [36]. These findings provided a biological rationale for including zinc sulphate as a reference treatment in the present study. However, these previous studies involved different animal species, experimental models, doses, and outcome measures and therefore do not establish 100 mg kg^−1^ day^−1^ as a validated positive-control dose for the present nicotine-exposed Wistar rat model. In the present study, zinc sulphate administered at 100 mg kg^−1^ day^−1^ did not produce the expected protective hormonal response. Therefore, the zinc-treated group should be interpreted cautiously and is more appropriately considered a reference treatment rather than a definitive positive control for androgenic protection. The absence of the expected response may reflect differences in species, toxicant exposure, treatment regimen, dose, treatment duration, or the specific reproductive outcomes assessed. Importantly, this finding does not indicate that zinc lacks reproductive or antioxidant activity in general, but rather that the selected zinc sulphate regimen did not provide an adequate pharmacological benchmark under the experimental conditions of the present study. Future studies should evaluate a model-specific zinc dose–response range and/or employ an established pharmacological positive control with demonstrated effects on reproductive and hypothalamic–pituitary–gonadal (HPG) axis outcomes.

The principal observation of this study is that oral gambir extract at 300 mg kg^−1^ day^−1^ (P2) significantly and consistently elevated all three HPG axis hormones relative to both the zinc positive control (POS; *p* < 0.05 for all three hormones) and the nicotine-only negative control (NEG; numerically superior). Critically, P2 FSH (11.20 ± 0.75 mIU mL^−1^) and testosterone (4.70 ± 0.26 ng mL^−1^) values exceeded those of the healthy controls (FSH = 9.86 mIU mL^−1^; testosterone = 3.99 ng mL^−1^), indicating that the gambir extract at this dose not merely prevented nicotine-induced hormonal suppression but actively augmented HPG axis function above basal physiological levels. This finding aligns with the recently reported androgenic-enhancing effects of catechin-rich green tea extracts by Musial et al. and with evidence that Nrf2/HMOX1 pathway activation in Leydig cells enhances steroidogenic capacity under oxidative stress conditions [7].

The non-monotonic dose–response pattern with P1 (100 mg kg^−1^) and P3 (500 mg kg^−1^) exhibiting intermediate, non-significant effects flanking the pharmacologically optimal P2 (300 mg kg^−1^) is consistent with a hormetic dose–response relationship, a phenomenon increasingly recognized for polyphenolic compounds [37,38]. Hormesis describes a biphasic dose–response in which low-to-moderate doses of a bioactive compound elicit beneficial adaptive responses through activation of cytoprotective transcription factors (Nrf2, FOXO) and endocrine signalling pathways, while higher doses exceeding the threshold for net antioxidant benefit may trigger pro-oxidant effects mediated by autoxidation of the catechol moiety, generating semiquinone radicals and superoxide anion through metal-catalyzed Fenton chemistry [37,38]. This pro-oxidant duality of polyphenols at supraphysiological concentrations has been documented in vitro [39] and may explain the attenuated androgenic response at 500 mg kg^−1^. However, it is equally plausible that the apparent attenuation at P3 reflects non-monotonic pharmacokinetics rather than pro-oxidant effects per se, as high concentrations of catechin may saturate intestinal absorption mechanisms (mediated by the sodium-dependent glucose transporter SGLT1 and the multidrug resistance protein MRP2) [32], reducing actual systemic bioavailability relative to the intermediate dose. Definitive mechanistic discrimination between these hypotheses requires dose-fractionation pharmacokinetic studies with plasma catechin quantification in parallel with in vivo hormonal outcome measurements.

The mechanistic basis for the androgenic augmentation observed at 300 mg kg^−1^ is multifactorial and hierarchically organized across the HPG axis. At the hypothalamic level, nicotine-induced neuroinflammation mediated by NF-κB-dependent IL-1β and TNF-α release from hypothalamic microglia suppresses kisspeptin/GnRH neuron activity and reduces pulsatile GnRH secretion [35]. Catechin’s predicted anti-inflammatory activity (PASS Pa = 0.645), potentially mediated through IKKβ inhibition and subsequent NF-κB suppression [40], may attenuate this neuroinflammatory suppression of GnRH neurons, restoring pulsatile gonadotrophin secretion and explaining the elevated FSH and LH observed in the P2 group. At the pituitary level, oxidative inactivation of gonadotroph GnRH receptors by nicotine-derived ROS is likely reversed by catechin’s direct radical scavenging activity and Nrf2/HMOX1-mediated upregulation of pituitary antioxidant defences [40]. At the testicular level, catechin’s membrane integrity agonism (Pa = 0.950) protects Leydig cell mitochondrial membranes from ROS-mediated peroxidative disruption, preserving the mitochondrial membrane potential (ΔΨm) required for the energy-dependent import of cholesterol by StAR and its subsequent conversion by CYP11A1 to pregnenolone, the committed step in testosterone biosynthesis [16]. HMOX1-generated CO and bilirubin within the testicular microenvironment further sustain mitochondrial integrity and provide a local antioxidant shield [7]. Finally, catechin may additionally exert direct steroidogenic effects through androgen receptor (AR)-independent mechanisms: flavonoids have been reported to stimulate cAMP synthesis in Leydig cells through inhibition of phosphodiesterase (PDE), potentiating the luteinizing hormone receptor (LHR)–adenylyl cyclase–protein kinase A (PKA) signaling cascade that drives StAR phosphorylation and acute steroidogenic activation [34].

Collectively, these mechanistic pathways provide a coherent and biologically plausible explanation for the observed HPG axis augmentation by gambir extract at 300 mg kg^−1^ and establish testable hypotheses for future mechanistic studies incorporating testicular CYP11A1 and StAR protein expression analysis, hypothalamic GnRH pulse characterization, and Leydig cell cAMP quantification. This study is limited by the absence of histopathological evaluation and pharmacokinetic validation, which should be addressed in future investigations.

All three gambir extracts demonstrated broad-spectrum inhibitory activity across Gram-positive bacteria (*S. aureus* & *C. acnes*), Gram-negative bacteria (*E. coli* & *S.* Typhi), and pathogenic fungi (*C. albicans* & *A. fumigatus*). The Halaban extract exhibited the greatest overall antibacterial potency, with the largest inhibition zones against *E. coli* (16.23 mm) and *S.* Typhi (14.12 mm), mirroring the hierarchy of antioxidant activity and catechin content and consistent with prior reports of catechin as the principal antimicrobial determinant in gambir [4,6]. The inhibition zone diameters recorded in this study compare favorably with those reported by Adamczak et al. for catechin against *E. coli* (12–18 mm) and *S. aureus* (10–15 mm) under comparable disc diffusion conditions, validating the external consistency of the present antimicrobial data [41].

The antimicrobial mechanisms of gambir extracts are multifactorial and organism-specific. Against Gram-positive bacteria, bioactive compounds disrupt the cytoplasmic membrane through hydrophobic insertion of their chromane scaffold and hydrogen bonding with membrane phospholipids, increasing membrane permeability and inducing cytoplasmic leakage of essential metabolites, including potassium ions and ATP [41]. Against Gram-negative organisms, the amphiphilic character of bioactive compounds facilitates interaction with lipopolysaccharide (LPS) components of the outer membrane through competitive displacement of structurally stabilizing divalent cations (Mg^2+^, Ca^2+^), leading to outer membrane destabilisation and enhanced intracellular accumulation of catechin [41,42]. At the intracellular level, bioactive compounds inhibit key bacterial metabolic enzymes—including topoisomerase, β-lactamase, and ATP synthase—through metal ion chelation and covalent protein modification, contributing to concentration-dependent bacteriostatic and bactericidal effects [42]. This multiplex mechanism of action is clinically significant in the context of antimicrobial resistance, as agents targeting multiple pathways simultaneously impose a lower probability of resistance emergence compared with single-target antibiotics [42].

The moderate antifungal activity against *C. albicans* and *A. fumigatus* is consistent with reports of polyphenol-mediated disruption of ergosterol biosynthesis and β-1,3-glucan-dependent cell wall integrity in fungal organisms [41,42]. Notably, the consistent inhibition of *C. acnes* (10.34–11.59 mm across all extracts) is of direct clinical relevance given the etiological role of this anaerobic Gram-positive organism in the pathogenesis of inflammatory acne vulgaris and the growing global burden of antibiotic-resistant *C. acnes* strains in dermatological practice [16]. The parallel between antimicrobial potency rankings and antioxidant and catechin content data further reinforces catechin as the primary bioactive determinant across multiple pharmacological endpoints in gambir.

The highest-ranked predicted activity, membrane integrity agonism (Pa = 0.950), has profound relevance to spermatozoal biology. Spermatozoa are uniquely vulnerable to oxidative membrane damage due to the exceptionally high docosahexaenoic acid (DHA; 22:6n-3) content of the sperm plasma membrane and the limited cytoplasmic volume available for antioxidant enzyme reserves [34,36]. Nicotine-derived ROS initiate phospholipid hydroperoxide cascades that ultimately compromise membrane fluidity, disrupt the acrosomal cap, and impair progressive motility [34]. The predicted capacity of catechin to function as a membrane integrity agonist is mechanistically plausible given its established ability to intercalate within lipid bilayers through hydrophobic interactions with the chromane ring system, effectively acting as a lipophilic radical chain-breaking agent within membrane microdomains [7]. This in silico prediction converges with in vitro evidence from Musial et al., who demonstrated that green tea catechins significantly attenuated 4-hydroxynonenal (4-HNE)-induced membrane lipid peroxidation in human sperm preparations [7].

The high predicted probability for HMOX1 induction (Pa = 0.778) is mechanistically significant. HMOX1 (hemoxygenase-1) is an Nrf2-regulated, stress-responsive enzyme that catabolizes heme to generate carbon monoxide (CO), biliverdin (subsequently reduced to the antioxidant bilirubin), and free iron (sequestered by ferritin) [7,40]. HMOX1 induction represents an amplified, second-tier cytoprotective response that extends well beyond the direct radical scavenging capacity of catechin itself. At the molecular level, catechin-mediated Nrf2 activation proceeds through electrophilic modification of Kelch-like ECH-associated protein 1 (Keap1) cysteine residues (particularly Cys151 and Cys288), disrupting the Keap1–Nrf2 interaction and facilitating nuclear translocation of Nrf2 and activation of antioxidant response element (ARE) genes [40]. This mechanism has been experimentally validated for catechin in murine Leydig cell models by Pervin et al. [40], lending direct biological relevance to the PASS prediction in the context of testicular steroidogenesis. The predicted TP53 enhancement (Pa = 0.826) and caspase-3 stimulation (Pa = 0.761) further suggest that catechin may facilitate selective elimination of irreparably oxidatively damaged spermatogenic cells—a quality control mechanism that, paradoxically, improves overall spermatogenic output by preventing propagation of genotoxically compromised cells [7].

## 4. Materials and Methods

### 4.1. Plant Material and Sample Procurement

*Uncaria gambir* Roxb. extracts (gambir) were procured from three principal production regions of West Sumatra, Indonesia: Mungka District and Halaban District (Limapuluh Kota Regency) and Koto XI Tarusan District (Pesisir Selatan Regency). All samples were obtained directly from certified local farmers as dry, commercially processed gambir blocks and were stored under ambient conditions (temperature < 25 °C, relative humidity < 60%) in sealed polyethylene bags, protected from light and moisture, until analysis.

### 4.2. Chemicals and Reagents

Analytical-grade ethanol, methanol, acetonitrile, and formic acid were obtained from Merck KGaA (Darmstadt, Germany). DPPH (2,2-diphenyl-1-picrylhydrazyl), ascorbic acid, gallic acid, quercetin, rutin hydrate, and (+)-catechin standards were purchased from Sigma-Aldrich (St. Louis, MO, USA). Dimethyl sulphoxide (DMSO) and Mueller–Hinton agar (MHA) were sourced from Oxoid (Basingstoke, UK). Sabouraud dextrose agar (SDA) was procured from HiMedia Laboratories (Mumbai, India). Ultrapure water (18.2 MΩ cm) was produced in-house using a Milli-Q purification system (Merck Millipore, Burlington, MA, USA). All solvents used for HPLC analysis were HPLC-grade.

### 4.3. Antioxidant Activity Determination (DPPH Radical Scavenging Assay)

Free radical scavenging activity was determined using the DPPH method as described by [43] with minor modifications. A stock solution of DPPH (0.2 mM) was freshly prepared in methanol and protected from light. Gambir extracts were dissolved in methanol at concentrations of 1.0, 2.0, 4.0, 6.0, 8.0, 10.0 and 12.0 µg mL^−1^. Aliquots (1.0 mL) of each extract concentration were mixed with an equal volume of DPPH solution in amber microtubes and vortexed briefly. The reaction mixtures were incubated at 25 ± 1 °C in the dark for 30 min. Absorbance was measured at 517 nm against a methanol blank using a UV–Vis spectrophotometer (Shimadzu UV-1800, Kyoto, Japan). Ascorbic acid served as the positive control. All measurements were performed in triplicate. Radical scavenging activity was expressed as percentage inhibition according to Equation (1):

(1) $$\%\;Inhibition=\;\frac{A_{0}-A_{1}}{A_{0}}\times 100$$

where A_0_ denotes the absorbance of the control (DPPH solution without extract) and A_1_ denotes the absorbance of the test solution (DPPH with extract). The IC_50_ value (concentration required to scavenge 50% of DPPH radicals) was determined by nonlinear logistic regression and expressed as µg mL^−1^, with the response constrained between the theoretical minimum of 0% and maximum of 100% [44,45]. A lower IC50 value indicates superior antioxidant potency [46].

### 4.4. HPLC-DAD Quantification of Catechin and Condensed Tannin

Quantitative analysis of catechin and condensed tannin was performed by high-performance liquid chromatography with diode-array detection (HPLC-DAD; Shimadzu Prominence LC-20A, Kyoto, Japan) following the procedure described in reference [12] with adaptations. Gambir samples (100 mg) were dissolved in methanol–water (7:3, *v*/*v*) and subjected to ultrasonication (30 min, 25 °C) before clarification by centrifugation (5000× *g*, 10 min) and filtration through 0.22 µm polytetrafluoroethylene (PTFE) syringe filters. Injection volume was 10 µL.

Chromatographic separation was achieved on an Agilent Zorbax SB-C18 column (250 × 4.6 mm, 5 µm particle size) maintained at 30 °C. The mobile phase consisted of (A) ultrapure water containing 0.1% formic acid (*v*/*v*) and (B) acetonitrile, delivered at a flow rate of 1.0 mL min^−1^ under the following gradient program: 0–5 min, 5% B; 5–20 min, 5–30% B; 20–30 min, 30–60% B; 30–35 min, 60–5% B; 35–40 min, 5% B (re-equilibration). Detection wavelength was 280 nm. Catechin and tannin contents were quantified against external calibration curves constructed from certified reference standards over the concentration range 1–100 µg mL^−1^, with linearity coefficients (R2) ≥ 0.999 [47].

### 4.5. Qualitative HPLC-DAD Detection of Selected Phenolic and Flavonoid Compounds

Selected phenolic and flavonoid compounds were qualitatively evaluated by HPLC-DAD using gallic acid, quercetin, and rutin hydrate as reference standards, following the method of Bondam et al. [48] with slight modifications. The analysis was intended to assess selected target compounds rather than provide a comprehensive characterization or quantitative profiling of the phenolic and flavonoid fractions. For sample preparation, 0.1 g of gambir sample was dissolved in methanol and made up to 10 mL, followed by sonication for 30 min. The extract was first filtered through Whatman No. 42 filter paper, and 1 mL of the filtrate was further diluted to 10 mL with methanol. The diluted solution was subsequently filtered through a 0.22 µm PTFE syringe filter and transferred into an HPLC vial for analysis. Chromatographic separation was performed using a C18 column (250 × 4.6 mm) with gradient elution using acetonitrile and 0.2% aqueous formic acid as mobile phases at a flow rate of 1 mL min^−1^. The column temperature was maintained at 30 °C, the injection volume was 5 µL, and detection was performed using a diode-array detector at 278 nm. Sample chromatograms were compared with the corresponding reference-standard chromatograms primarily based on retention time under identical analytical conditions. Because the method was not quantitatively validated for these target compounds and direct retention-time correspondence was not consistently observed between the sample peaks and the reference standards, the results were interpreted as a qualitative chromatographic assessment rather than as definitive compound identification or quantitative determination [49].

### 4.6. Antimicrobial Activity Assessment

Antimicrobial activity was evaluated by the Kirby–Bauer disc diffusion method in accordance with Clinical and Laboratory Standards Institute (CLSI) guidelines (M02, 13th ed.) [39]. Six reference strains were utilised: *Escherichia coli* (ATCC 25922), *Staphylococcus aureus* (ATCC 25923), *Cutibacterium acnes* (ATCC 6919), *Salmonella enterica* serovar Typhi (ATCC 14028), *Candida albicans* (ATCC 10231), and *Aspergillus fumigatus* (ATCC 16404).

Gambir extracts were dissolved in DMSO at 100 mg mL and sterilized by filtration through 0.22 µm PTFE membranes. Sterile paper discs (6 mm diameter; Whatman No. 1) were impregnated with 10 µL of extract solution and air-dried at ambient temperature. Mueller–Hinton agar (MHA) was used for bacterial strains and Sabouraud dextrose agar (SDA) for fungal strains; both media were inoculated with standardized suspensions (0.5 McFarland standard, equivalent to ~1.5 × 108 CFU mL). Incubation conditions were 37 °C for 24 h (bacteria) and 28 °C for 48–72 h (fungi). Inhibition zone diameters (inclusive of disc diameter) were measured in millimetres using a digital calliper. Ciprofloxacin (5 µg disc) and fluconazole (25 µg disc) served as positive controls; DMSO served as the negative control. All assays were conducted in triplicate.

### 4.7. PASS Biological Activity Prediction

Computational prediction of the biological activity spectrum of catechin was performed using PASS Online (Prediction of Activity Spectra for Substances; http://www.way2drug.com/PASSonline, accessed on 18 July 2026), an established structure–activity relationship (SAR)-based platform trained on a curated dataset of more than 250,000 biologically characterized compounds [50,51]. The catechin molecular structure (PubChem CID: 9064) was retrieved from the PubChem database [52]. Activity was reported as the probability of activity (Pa) and probability of inactivity (Pi); compounds with Pa > 0.7 were considered to have a high likelihood of activity, 0.5 ≤ Pa ≤ 0.7 moderate, and Pa < 0.5 low [53]. Biological activities relevant to antioxidant defense, membrane protection, and reproductive endocrine function were selectively evaluated and reported.

### 4.8. ADMET Profiling via SwissADME

Pharmacokinetic and drug-likeness properties of catechin were computed using the SwissADME web server (http://www.swissadme.ch, accessed on 18 July 2026) developed by the Molecular Modelling Group of the Swiss Institute of Bioinformatics [54]. Parameters evaluated included molecular weight, consensus LogP (iLOGP, XLOGP3, WLOGP, MLOGP, SILICOS-IT), hydrogen bond donors (HBDs) and acceptors (HBAs), topological polar surface area (TPSA), number of rotatable bonds, Lipinski’s rule of five compliance, gastrointestinal (GI) absorption, blood–brain barrier (BBB) permeability, P-glycoprotein substrate potential, cytochrome P450 (CYP1A2, CYP2C19, CYP2C9, CYP2D6, CYP3A4) inhibitory profiles, PAINS (Pan-Assay Interference Compounds) alerts, and bioavailability score assessed via the boiled-egg model [30,31]. SMILES notation for catechin was obtained from PubChem [31].

### 4.9. Gambir Extract Preparation and In Vivo Experimental Design

The gambir sample used for the in vivo experiment was obtained directly from a local farmer in Halaban, Lima Puluh Kota Regency, West Sumatra, Indonesia. The extract administered to the rats was the Halaban gambir extract (*U. gambir* var. Mancik; GenBank accession MZ927015), designated as laboratory sample GHA. The same sample was used for catechin analysis and preparation of the extract administered to the animals. Gambir was macerated with ethanol at a 1:3 (*w*/*v*) sample-to-solvent ratio for 2 × 24 h, followed by filtration and concentration using a rotary evaporator at 40 °C. The extraction yield was 14.6%, and the catechin content of the GHA sample was 7.91 ± 0.07 mg g^−1^, as determined by HPLC-DAD. The resulting extract was used for the animal experiment, with doses of 100, 300, and 500 mg kg^−1^ day^−1^ calculated based on the obtained extract. Before administration, the extract was dispersed in distilled water and administered orally by gavage.

All animal procedures were performed in accordance with international guidelines for the care and use of laboratory animals and were approved by the Institutional Animal Ethics Committee of Baiturrahmah University (Approval No.:015/ETIK-FKUNBRAH/03/05/2025). Male Wistar rats (*Rattus norvegicus*, 200–250 g, 10–12 weeks) were obtained from a certified supplier, housed under controlled environmental conditions (22 ± 2 °C, 50 ± 10% relative humidity, 12 h light/dark cycle), and provided with standard pelleted diet and water ad libitum.

The gambir extract doses of 100, 300, and 500 mg kg^−1^ day^−1^ were selected to provide a graded dose range for evaluating the dose–response pattern in nicotine-exposed rats. Previous experimental studies have evaluated gambir-containing preparations across oral dose ranges of approximately 100–400 mg kg^−1^, while a recent study in Sprague–Dawley rats specifically evaluated *U. gambir* at 200, 300, and 400 mg kg^−1^ [55]. Based on this evidence, 100 mg kg^−1^ day^−1^ was selected as a lower exploratory dose, 300 mg kg^−1^ day^−1^ as an intermediate dose within the previously investigated range, and 500 mg kg^−1^ day^−1^ as a higher exploratory dose extending beyond the commonly investigated range. The three doses were therefore selected to explore the dose–response pattern rather than to represent previously established therapeutic doses.

Six rats were allocated to each experimental group (n = 6 per group). The sample size was selected with reference to previous animal studies using comparable experimental designs and outcome measures, while also considering the ethical principle of minimizing animal use and the available experimental resources. No a priori power analysis was performed before the experiment; therefore, the present sample size should be considered an empirically informed design choice rather than a sample size derived from a predefined statistical power calculation.

Following a one-week acclimatization period, 36 rats were randomly allocated to six experimental groups (n = 6 per group) as follows: positive group: nicotine 1.0 mg/kg/day + zinc sulphate 100 mg/kg/day (oral); negative group: nicotine 1.0 mg kg/day/ + oral vehicle; control: healthy rats and no nicotine daily; P1 nicotine 1.0 mg/kg/day/ + gambir extract 100 mg/kg/day (oral); P2 nicotine 1.0 mg/kg/day/ + gambir extract 300 mg/kg/day (oral) and P3 nicotine 1.0 mg/kg/day/ + gambir extract 500 mg/kg/day (oral) for 30 days.

Nicotine hydrogen tartrate was dissolved in physiological saline and administered by subcutaneous injection once daily. At the end of the 30-day treatment period, rats were anaesthetized with ketamine (80 mg kg^−1^) and xylazine (10 mg kg^−1^, intramuscular), blood was collected by cardiac puncture, and serum was separated by centrifugation (3000× *g*, 15 min, 4 °C). Serum concentrations of FSH, LH, and testosterone were determined by enzyme-linked immunosorbent assay (ELISA) using commercially validated rat-specific kits (CUSABIO, Houston, TX, USA) according to the manufacturer’s instructions.

### 4.10. Statistical Analysis

All quantitative data are presented as mean ± standard deviation (SD). Prior to one-way analysis of variance (ANOVA), the normality of data distribution was assessed using the Shapiro–Wilk test, while the homogeneity of variances was evaluated using Levene’s test. Variables satisfying the assumptions of normality and homogeneity of variance were analysed using one-way ANOVA, followed by Tukey’s honestly significant difference (HSD) post hoc test for pairwise comparisons. Pearson’s product–moment correlation coefficient (r) was used to assess the relationship between phytochemical contents (catechin and condensed tannin) and antioxidant activity expressed as DPPH IC_50_. Statistical significance was set at *p* < 0.05. All statistical analyses were performed using IBM SPSS Statistics, version 26.0 (IBM Corp., Armonk, NY, USA) [37,38]. Superscript letters in the tables indicate statistically homogeneous subsets determined by Tukey’s HSD post hoc test.

## 5. Conclusions

This integrated in vitro, in silico, and in vivo study provides evidence that *Uncaria gambir* Roxb. extract exhibits antioxidant activity and may modulate reproductive endocrine responses in nicotine-exposed male rats due to its richness in multiple phenolic compounds, including catechin. Among the regional extracts, Halaban showed the strongest antioxidant activity, with an estimated DPPH IC_50_ of 6.99 ± 0.28 µg mL^−1^. In the in vivo study, the 300 mg kg^−1^ day^−1^ dose produced the strongest overall response in serum FSH, LH, and testosterone. In contrast, the 500 mg kg^−1^ day^−1^ dose provided no additional benefit, suggesting a non-linear dose–response pattern. The zinc sulphate-treated group did not show the expected protective hormonal response and was therefore considered a reference treatment rather than a definitive positive control. These findings support further investigation of standardized gambir extracts using prospectively justified sample sizes and model-specific dose–response, pharmacokinetic, and mechanistic studies.

## Figures and Tables

**Table 1:** Antioxidant activity (IC_50_) and HPLC-DAD-quantified phytochemical composition of gambir extracts from three West Sumatran production regions.

Production Region	Antioxidant IC_50_ (µg mL^−1^)	Catechin (mg g^−1^)	Condensed Tannin (mg g^−1^)
Halaban	6.99 ± 0.28 ^a^	7.91 ± 0.07 ^c^	14.31 ± 0.21 ^b^
Mungka	9.50 ± 0.13 ^c^	6.22 ± 0.57 ^b^	14.45 ± 0.92 ^b^
Pesisir Selatan	8.49 ± 0.07 ^b^	3.59 ± 0.63 ^a^	10.41 ± 0.84 ^a^

Values are expressed as mean ± SD (n = 3). Different superscript letters within columns indicate statistically significant differences (Tukey’s HSD post hoc test, *p* < 0.05). IC_50_ = inhibitory concentration required to scavenge 50% of DPPH radicals.

**Table 2:** Pearson correlation between phytochemical content and antioxidant IC_50_ (µg mL^−1^).

Variables Compared	Pearson r	*p*-Value	Interpretation
Catechin vs. IC_50_	−0.489	0.675	Moderate negative (NS)
Tannin vs. IC_50_	−0.082	0.948	Negligible (NS)

NS = not statistically significant (*p* > 0.05). Analyses limited to n = 3 regional samples; statistical power is insufficient to draw definitive conclusions.

**Table 3:** Inhibition zone diameters (mm) of gambir extracts and controls against six reference microorganisms.

Region	*E. coli* (mm)	*S. aureus* (mm)	*C. acnes* (mm)	*S.* Typhi (mm)	*C. albicans* (mm)	*A. fumigatus* (mm)
Halaban	16.23	12.21	11.46	14.12	11.59	6.21
Mungka	15.08	11.21	11.59	13.34	9.22	7.87
Pesisir Selatan	14.34	11.59	10.34	12.89	14.34	5.11
Ampisilin (10 µg/disc)	15.58	12.46	12.21	18.37	NA	NA
Voriconazole (1 µg/dsic)	NA	NA	NA	NA	17.83	21.23
DMSO	0.00	0.00	0.00	0.00	0.00	0.00

Values represent mean inhibition zone diameters (mm) inclusive of disc diameter (6 mm); n = 3 independent replicates. Ampicillin (10 µg/disc) and voriconazole (1 µg/disc) served as positive controls for antibacterial and antifungal assays, respectively. NA = Not applicable.

**Table 4:** PASS-predicted biological activities of catechin relevant to antioxidant defense, membrane protection, and reproductive endocrine function.

Predicted Biological Activity	Pa	Pi
Membrane integrity agonist	0.950	0.004
TP53 expression enhancer	0.826	0.009
HMOX1 expression enhancer	0.778	0.004
Caspase-3 stimulant	0.761	0.008
Lipid peroxidase inhibitor	0.692	0.005
Anti-inflammatory	0.645	0.023
Free radical scavenger	0.633	0.005
Antioxidant	0.587	0.005

Pa = probability of activity; Pi = probability of inactivity. Activities with Pa > 0.70 are classified as high-probability, 0.50–0.70 as moderate-probability.

**Table 5:** SwissADME-predicted pharmacokinetic and drug-likeness parameters of catechin (PubChem CID: 9064).

Parameter	Value	Interpretation
Molecular Formula	C_15_H_14_O_6_	Polyphenolic flavan-3-ol
Molecular Weight	290.27 Da	Drug-like (<500 Da)
H-bond Donors (HBDs)	5	Lipinski-compliant (≤5)
H-bond Acceptors (HBAs)	6	Lipinski-compliant (≤10)
Rotatable Bonds	1	High rigidity; low conformational entropy loss
TPSA	110.38 Å^2^	Favorable for oral absorption (<140 Å^2^)
Consensus LogP	−0.48	Moderately hydrophilic; rapid plasma distribution
GI Absorption	High	Efficient small intestinal uptake predicted
BBB Permeability	No	Limited CNS penetration (advantageous: peripheral target)
CYP Inhibition	None predicted	Low DDI risk across all major isoforms
Lipinski Rule of Five	0 violations	Full drug-like chemical space compliance
Bioavailability Score	0.55	Moderate oral bioavailability
PAINS Alert	1 (catechol)	Redox activity = mechanism, not artefact

BBB = blood–brain barrier; CYP = cytochrome P450; DDI = drug–drug interaction; GI = gastrointestinal; PAINS = Pan-Assay Interference Compounds; TPSA = topological polar surface area.

**Table 6:** Serum FSH, LH, and testosterone concentrations following 30-day treatment in nicotine-challenged male Wistar rats.

Group	Treatment	FSH (mIU mL^−1^)	LH (mIU mL^−1^)	Testosterone (ng mL^−1^)
HC	Healthy control (vehicle only)	9.86 ± 1.06 ^ab^	7.22 ± 0.54 ^ab^	3.99 ± 0.62 ^b^
NEG	Nicotine 1.0 mg kg^−1^ day^−1^	8.89 ± 2.79 ^ab^	7.69 ± 2.06 ^ab^	3.81 ± 1.08 ^ab^
POS	Nicotine + zinc sulphate 100 mg kg^−1^	7.02 ± 2.17 ^a^	6.38 ± 1.87 ^a^	2.69 ± 0.52 ^a^
P1	Nicotine + Gambir 100 mg kg^−1^	8.85 ± 0.70 ^ab^	7.84 ± 0.37 ^ab^	3.68 ± 0.15 ^ab^
P2	Nicotine + Gambir 300 mg kg^−1^	11.20 ± 0.75 ^b^	8.89 ± 0.30 ^b^	4.70 ± 0.26 ^b^
P3	Nicotine + Gambir 500 mg kg^−1^	8.26 ± 1.75 ^ab^	7.27 ± 0.90 ^ab^	3.55 ± 0.84 ^ab^

Values are expressed as mean ± SD (n = 6 per group). Different superscript letters within columns denote statistically significant differences (Tukey’s HSD, *p* < 0.05). HC = healthy control; NEG = nicotine-only (negative control); POS = nicotine + zinc sulphate (positive control); P1–P3 = nicotine + gambir extract at 100, 300, and 500 mg kg^−1^ day^−1^, respectively; FSH = follicle-stimulating hormone, LH = luteinizing hormone.

## Data Availability

All data generated or analyzed during this study are included in this published article.

## Supplementary Materials

The following supporting information can be downloaded at: https://www.mdpi.com/article/10.3390/ijms27177533/s1.

## Abbreviations

The following abbreviations are used in this manuscript:

4-HNE	4-hydroxynonenal
ADMET	Absorption, Distribution, Metabolism, Excretion, and Toxicity
ANOVA	analysis of variance
AR	androgen receptor
ARE	antioxidant response element
BBB	blood–brain barrier
CLSI	Clinical and Laboratory Standards Institute
CO	carbon monoxide
DAD	Diode-Array Detector
DMSO	Dimethyl Sulfoxide
DPPH	2,2-Diphenyl-1-picrylhydrazyl
ELISA	Enzyme-Linked Immunosorbent Assay
FSH	follicle-stimulating hormone
GI	gastrointestinal
GPx	glutathione peroxidase
HAT	hydrogen atom transfer
HBA	hydrogen bond acceptors
HBD	hydrogen bond donors
HMOX1	heme oxygenase-1
HPG	hypothalamic–pituitary–gonadal
HSD	Honestly Significant Difference
IC_50_	Half-Maximal Inhibitory Concentration
LC	Liquid Chromatography
LH	luteinizing hormone
LHR	luteinizing hormone receptor
MHA	Mueller–Hinton agar
NF-κB	Nuclear Factor Kappa B
Nrf2	nuclear factor erythroid 2-related factor 2
PAINS	Pan-Assay Interference Compounds
PDE	phosphodiesterase
PKA	protein kinase A
PTFE	polytetrafluoroethylene
PUFA	polyunsaturated fatty acid
ROS	reactive oxygen species
SAR	structure–activity relationship
SDA	Sabouraud dextrose agar
SET	single electron transfer
SOD	superoxide dismutase
TPSA	topological polar surface area
