# Supplementary material for: Hormetic Antioxidant Effects of Uncaria gambir on Male Reproductive Function: A Comprehensive Experimental and Computational Analysis
Source: Int J Mol Sci. 2026 Aug 22;27(17):7533. doi: 10.3390/ijms27177533 (PMC13565616; doi:10.3390/ijms27177533)
Supplement: Supplementary file 1 [file ijms-27-07533-s001.zip › ijms-4476199-supplementary.pdf]

Supplementary Figure S1

Supplementary Figure S1

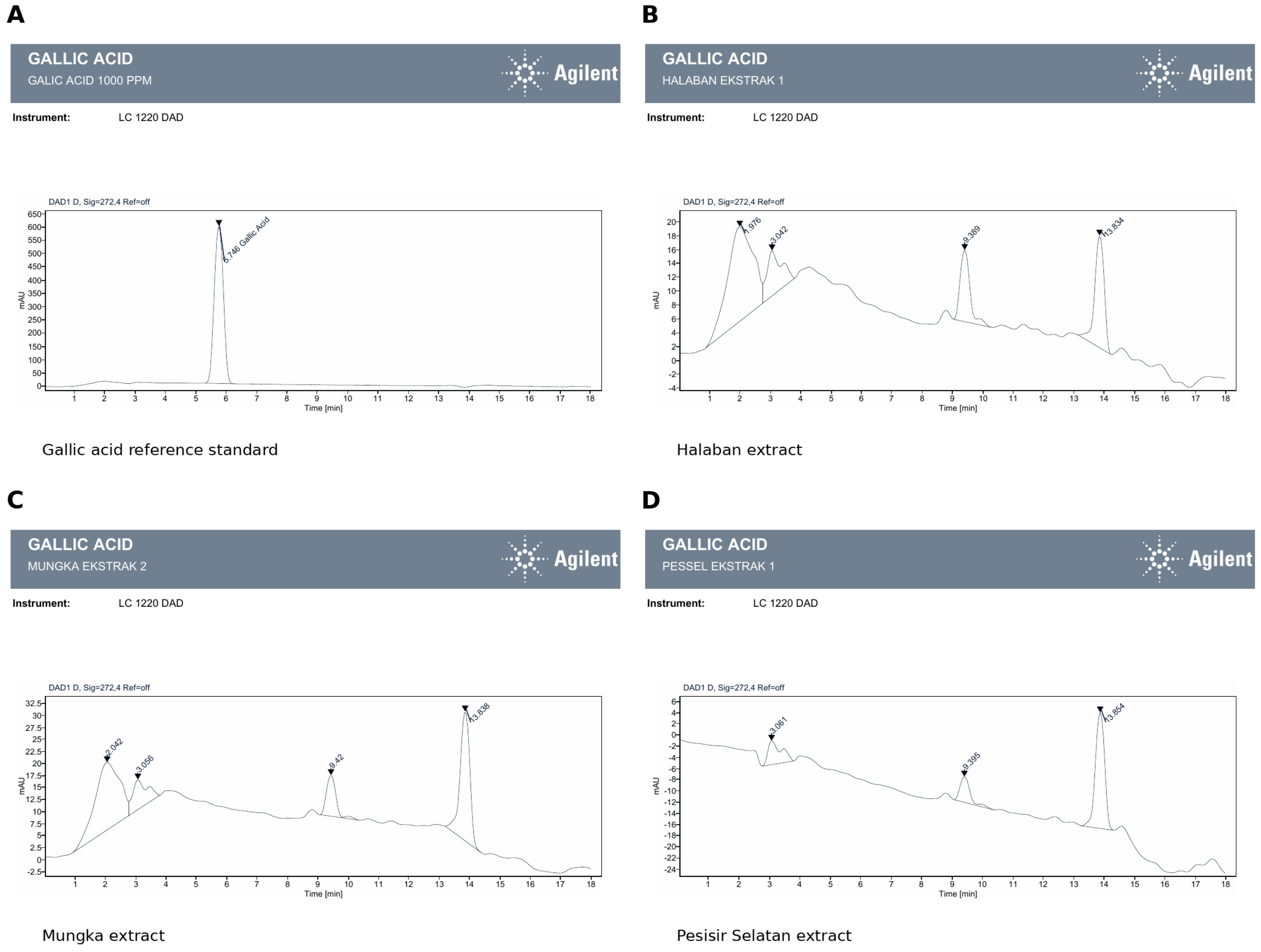

Figure S1. HPLC-DAD chromatograms of gallic acid reference standard and representative gambir extracts. (A) Gallic acid reference standard (retention time, RT = 5.746 min); (B) Halaban extract; (C) Mungka extract; and (D) Pesisir Selatan extract. Representative sample peaks and their retention times are indicated in the chromatograms. The sample profiles did not show direct retention-time correspondence with the gallic acid reference standard and therefore do not permit conclusive assignment of gallic acid to the observed sample peaks.

Supplementary Figure S2

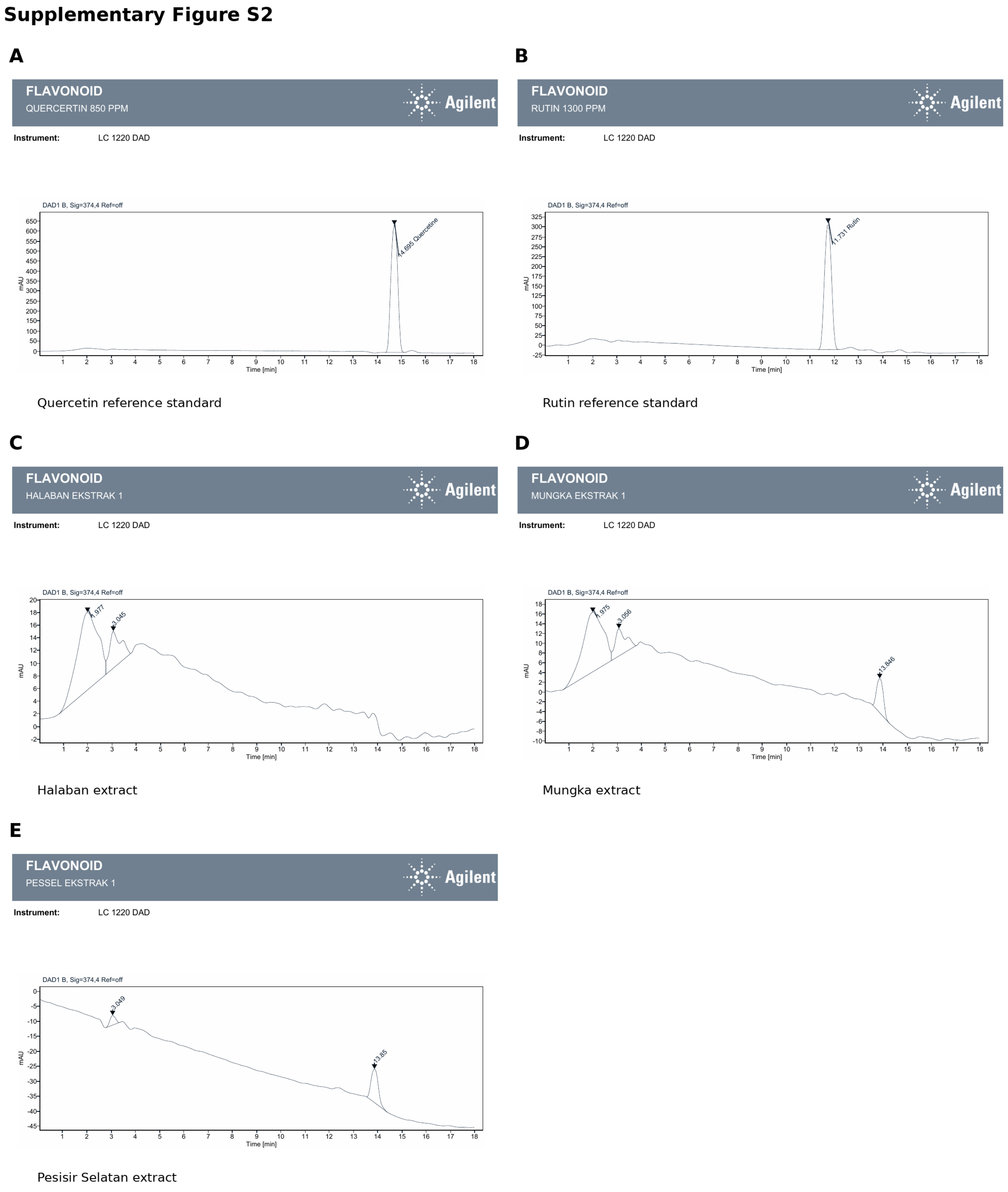

Figure S2. HPLC-DAD chromatograms of quercetin and rutin reference standards and representative gambir extracts. (A) Quercetin reference standard (RT = 14.695 min); (B) Rutin reference standard (RT = 11.731 min); (C) Halaban extract; (D) Mungka extract; and (E) Pesisir Selatan extract. Representative sample peaks and their retention times are indicated in the chromatograms. The sample profiles did not show direct retention-time correspondence with the respective reference standards and therefore do not permit conclusive assignment of quercetin or rutin to the observed sample peaks.
